# Dietary total antioxidant capacity is inversely associated with depression, anxiety and some oxidative stress biomarkers in postmenopausal women: a cross-sectional study

**DOI:** 10.1186/s12991-019-0225-7

**Published:** 2019-03-19

**Authors:** Maryam Abshirini, Fereydoun Siassi, Fariba Koohdani, Mostafa Qorbani, Hadis Mozaffari, Zahra Aslani, Mahshid Soleymani, Mahdieh Entezarian, Gity Sotoudeh

**Affiliations:** 1 Department of Community Nutrition, School of Nutritional Sciences and Dietetics, Tehran University of Medical Sciences, Hojatdost Street, Naderi Street, Keshavarz Blv., Tehran, Iran; 2 Students’ Scientific Research Center, Tehran University of Medical Sciences, Tehran, Iran; 3 Department of Cellular, Molecular Nutrition, School of Nutritional Sciences and Dietetics, Tehran University of Medical Sciences, Tehran, Iran; 4 Non-communicable Diseases Research Center, Alborz University of Medical Sciences, Karaj, Iran; 5 Chronic Diseases Research Center, Endocrinology and Metabolism Population Sciences Institute, Tehran University of Medical Sciences, Tehran, Iran

**Keywords:** Total antioxidant capacity, Depression, Oxidative stress, Menopause

## Abstract

**Background:**

Postmenopausal women are at higher risk of mental disorders. Oxidative stress has implication in the development of these disorders. Dietary total antioxidant capacity (DTAC) has been proposed as a tool for assessing dietary antioxidants intake. The relationship between DTAC with depression, anxiety and stress has not been investigated in postmenopausal women. Thus, we aimed to assess the association between DTAC and depression, stress and anxiety as well as oxidative stress biomarkers.

**Methods:**

This cross-sectional study was carried out on 175 postmenopausal women. Data on dietary intake and mental health were collected by 147-item semi-quantitative food frequency questionnaires (FFQ) and Depression Anxiety Stress Scales (DASS-42), respectively. Dietary and serum total antioxidant capacity (TAC), malondialdehyde (MDA), oxidized-LDL, and superoxide dismutase (SOD) were measured. ANOVA test was applied to compare the mean of variables across the tertiles of DTAC. The relationship between DTAC and oxidative stress biomarkers was determined through ANCOVA method. Simple and multivariate linear regression tests were performed to measure the relationship between DTAC and mental health.

**Results:**

Serum MDA level was significantly lower in the subjects at the highest tertiles of DTAC (*P*-value < 0.001). In addition, serum TAC level was significantly higher in subjects at the second tertile of DTAC (*P*-value = 0.04). DTAC was inversely and independently related to depression (β = − 0.16, *P*-value = 0.03) and anxiety scores (β = − 0.21, *P*-value = 0.007). There was no significant association between DTAC and stress score (β = − 0.10, *P*-value = 0.1).

**Conclusion:**

An inverse relationship was found between DTAC with depression, anxiety scores and some oxidative stress biomarkers in postmenopausal women. These findings indicate DTAC may be used for developing effective dietary measures for reducing depression and anxiety in these women.

## Introduction

Mental disorders have been associated with a wide range of chronic diseases, disability, and even mortality, particularly among elderly. Depression will be the second leading cause of disease in 2020 as reported by world health organization (WHO) [1]. Postmenopausal women are often at high risk of depression due to the lower level of estrogen [2], as data from longitudinal studies and meta-analyses have noted the chance of developing the depressive symptoms substantially increases during the menopausal transition and early postmenopausal years [3, 4]. Estrogen is involved in mood regulation, and its action is mediated through modulating the receptor binding of serotonin and noradrenaline as well as availability of these neurotransmitters at the synapsis places. So, the decline of estrogen during menopause may underlie depressive mood in menopausal women [5]. Additionally, because of estrogen decline, menopausal women face various distressing symptoms such as hot flashes and sleep disturbance which may increase their vulnerability for mood disorders [6]. Considering the high prevalence of depression (44.2%) and anxiety (67%) among postmenopausal women living in Tehran, Iran [7], finding an approach to modify these symptoms among the women during menopause is of vital importance.

Menopausal women are at high risk for oxidative stress mainly due to estrogen deprivation [8]. Existing evidence suggests that oxidative stress might be linked with development of menopause-associated depression. Estradiol (E_2_), a form of estrogen, exerts antioxidant effect through its chemical structure containing the phenolic ring [9]. Besides, E_2_ can confer antioxidant activity through modulating the gene expression and function of antioxidant enzymes such as superoxide dismutase (SOD), glutathione peroxidase (GPx) and catalase [10]; thereby, reduction in estrogen level may increase the oxidative stress. It was indicated that plasma lipoperoxide (LPO) levels, a marker of oxidative damage, were increased; while total antioxidant status (TAS) and activity of antioxidant enzymes were reduced in postmenopausal women compared with premenopausal women [8, 11].

Diet is considered as the main part of lifestyle modification [12]. For decades, the association between special dietary factors, particularly fruits and vegetables and mental health has been investigated [13]. Although fruits and vegetables have abundant amounts of dietary antioxidants, they cannot adequately represent the overall ability and interactions of antioxidants in the whole diet. Thus, the dietary concept which takes the whole dietary antioxidants into account has become more prominent. Dietary total antioxidant capacity (DTAC) is an indicator of diet quality which is used to estimate the cumulative power of antioxidants in the whole diet [14]. Recently, DTAC is described as an effective tool for determining the health outcomes in middle-aged and elderly populations [15].

To date, DTAC has been associated with various chronic diseases [16–18], but little attention has been paid to DTAC in relation to mental health. We are aware of a case–control study which showed no significant difference of DTAC between depressed and normal subjects [19]. Examining the relationship between DTAC and mental health is particularly relevant for Middle East countries, where the prevalence of mental disorder is rampant and the quality of life is not desirable. Moreover, concerning the studies that linked the mental disorders to increased risk of illnesses and mortality among the elderly [1], investigating the association between DTAC and mental health in postmenopausal women can be helpful. Given the limited knowledge, we aimed to elucidate the association of DTAC with depression, stress and anxiety and some oxidative stress in postmenopausal women.

## Methods

### Participants and study design

The details of the study procedure have been described previously [20]. In brief, subjects were 175 postmenopausal women that postmenopausal status identified by an absence of a menstrual cycle for at least 1 year. Participants were selected randomly from municipality health houses and health centers affiliated to Tehran University of Medical Sciences. Using random sampling method, two regions from six southern regions of Tehran were randomly selected. Of these two regions, ten urban health centers affiliated to Tehran University of Medical Sciences and ten health houses affiliated with the southern municipality of Tehran were randomly selected. Finally, this study was composed of 175 postmenopausal women up to age 76 years, who enrolled through attendance to urban health centers and health houses.

Subjects were recruited from September 2016 to Januarys 2017. Exclusion criteria were; obesity [body mass index (BMI) ≥ 40 kg/m^2^], medically diagnosed of chronic diseases including cancer, diabetes, stroke, multiple sclerosis, dementia, hyper or hypothyroidism. Women were excluded if they were alcohol consumer, smoker (smoking for at least once a week), currently on hormone therapy or in the preceding 6 months or had any modifications in habitual diets. This study was approved by the Ethics Committee of Tehran University of Medical Sciences. Written informed consent was obtained from all subjects.

### Dietary intake assessment

Subjects interviewed to respond to the validated semi-quantitative food frequency questionnaire (FFQ) consisted of 147 items to assess the frequency of food consumption of participants during the previous year [21]. Each subject’s dietary data were converted to the gram, and then the average daily intake of nutrients and total energy were determined according to the Nutritionist 4 software modified for Iranian foods.

DTAC was calculated by summing the individual dietary antioxidant capacities based on the oxygen radical absorbance capacity (ORAC) as described in our previous studies [20, 22]. The DTAC values of the individuals were reported as micromole of Trolox Equivalents per day (µmol TE/day).

### Sociodemographic, anthropometrics and physical activity measurement

Self-reported information on general characteristics including age, educational level, marital and economic status, time since menopause, medical history, medicine and nutritional supplement use were obtained from all women. Height was measured using the wall-mounted stadiometer to the nearest 0.5 cm. Weight was measured in light clothing to the nearest 0.1 kg with a digital weighing scale (Seca725 GmbH & Co. Hamburg, Germany). Waist circumference (WC) was assessed to the nearest 0.5 cm using the flexible tape at the midpoint between the lowest rib and the iliac crest. BMI was calculated by dividing body weight (kg) by the square of body height (m^2^). Physical activity was measured using the short form of the International Physical Activity Questionnaire (IPAQ) [23].

### Blood collection and plasma biomarker processing

12–14-h fasting blood sample was drawn and collected in a tube without ethylenediaminetetraacetic acid (EDTA) or heparin and placed on ice for 1 h. Then, it was centrifuged at 3000*g* for 10 min. Serum was separated, and frozen at − 80 °C for various biochemical evaluations. Serum total antioxidant capacity (TAC) was analyzed by a commercially available Kit (ZellBio GmbH, Germany) in accordance with the instructions of the manufacturer, based on the colorimetric assay of oxidation reduction at the wavelength of 490 nm. Intra- and inter-assay coefficients of variation (CVs (were less than 3.4% and 4.2%, respectively. Malondialdehyde (MDA) was measured by commercially available colorimetric assay kit (ZellBio GmbH, Germany) according to the instructions of the manufacturer. In this method, MDA reacts with thiobarbituric acid (TBA) under a high temperature, then MDA–TBA produced by this reaction is measured based on the colorimetric assay on an acidic medium and high temperature (90–100 °C) at 535 nm. Intra- and inter-assay CVs were 5.8% and 7.6%, respectively. Superoxide dismutase (SOD), were determined using commercially available kits (ZellBio GmbH, Germany) based on the colorimetric method (420 nm), with intra- and inter-assay CVs of 5.8% and 7.2%, respectively. Oxidized low-density lipoprotein (Ox-LDL) was measured using an ELISA kit (ZellBio GmbH, Germany), with intra- and inter-assay CVs less than 10% and 12%, respectively.

### Mental health assessment

Depression Anxiety Stress Scales (DASS-42) was applied for measuring the mental health status of subjects. The DASS-42 consists of 42 items, 14 items per subscale: depression, anxiety, and stress. The respondent scores each item based on frequency or severity of emotional experiences over the last week on four-point scale ranged from 0 to 3. The score of 0 considered that the item “no symptoms at all” to 3 that indicates the item was “very severe”. Five categories are represented for depression including normal (sore less than 9), mild (10–13), moderate (14–20), severe (21–27) and very severe (higher than 27). Based on obtained scores for stress, the following categories are considered: normal (less than 14), mild (15–18), moderate (19–25), severe (26–33) and very severe (higher than 33). The total scores for the anxiety subscale are categorized as normal (0–7), mild (8–9), moderate (10–14), severe (15–19) and extremely severe (higher than 19) [24]. This DASS-42 scale was tested on non-clinical samples to measure its psychometric properties [25, 26]. The validity and reliability of DASS-42 questionnaire have been assessed in Iranian subjects previously, and all three subscales were found to have high Cronbach’s alpha coefficients (0.94 for the depression, 0.87 for the stress and 0.85 for the anxiety) [27].

### Statistical analysis

SPSS software version 16 (Inc. Chicago, IL) was applied to carry out all analyses. Parameters were checked for normality by the Kolmogorov–Smirnov test, and data were reported as mean ± standard deviation and median (interquartile range) for normal and non-normal distribution, respectively. DTAC was adjusted for energy intake through the residual method [28]. Comparison of participant characteristics across the tertiles of DTAC was made by ANOVA test for continuous variables and chi-square test for categorical variables. Partial correlation adjusted for energy intake was performed to assess the correlation of DTAC and serum TAC with some dietary antioxidant nutrients. Adjusted means of oxidative stress biomarkers across the tertiles of DTAC were compared using the analysis of covariance (ANCOVA). The association between oxidative stress biomarkers and DTAC was determined by multiple linear regression. In addition, multiple linear regressions were performed to evaluate the association of DTAC and log-transformed psychological factors in three sets of models: (1) unadjusted model; (2) adjusted for age and time since menopause; (3) model 2 further adjusted for education, WC, physical activity, dietary supplement use, dietary intake of fiber, energy, and coffee. These covariates have been shown to be associated with DTAC in the present and previous studies [14, 29, 30].

## Results

The distribution of women in the subscale category of mental health showed that 15% of women had severe and very severe symptoms of anxiety, while this distribution for depression and stress was 7% and 5%, respectively. Furthermore, our data revealed that about 4% of the population study (seven women) had severe and very severe symptoms of all three disorders (data not shown).

As shown in Table 1, with respect to general characteristics, no significant difference was observed across the tertiles of DTAC. The subject at higher tertiles of DTAC had higher dietary supplement use; however, it was not statistically significant (P-trend = 0.08). Women included in the higher tertiles of DTAC had higher median WC than women included in the lower tertile of DTAC (106 vs. 103 cm), although the association was not statistically significant. In addition, subjects in higher tertiles of DTAC were more likely to be physically active (*P*-trend = 0.02). Although DTAC was not associated with intake of energy, carbohydrate, and protein, but it was inversely associated with fat intake, (P-trend = 0.01), percent of energy from fat (P-trend = 0.003) and positively associated with percent of energy from carbohydrate (P-trend < 0.001). In terms of psychological factors, our data revealed that women at the higher tertiles of DTAC had fewer symptoms of depression and anxiety (P-trend < 0.04).

**Table 1:** Demographic, anthropometric, dietary and psychological characteristics of study participant across the tertiles of energy-adjusted DTAC (*n *= 175)

Characteristics	Tertiles of energy-adjusted DTAC			*P*-trend**
	T1 (*n *= 58)	T2 (*n *= 59)	T3 (*n *= 58)	
Energy-adjusted DTAC, median (μmol/day)	14,262.79	18,462.49	23,208.09	
*Sociodemographic factors*				
Age (year)^†^	56.0 (8.2)	55.0 (7.0)	57.0 (10.0)	0.4
Education (year)^†^	4.0 (6.0)	5.0 (6.0)	5.0 (6.0)	0.1
Menopausal years (year)^†^	6.5 (9.7)	4.0 (6.0)	8.0 (11.0)	0.9
Marital status n (%)*				
Married	45 (25.7)	51 (29.1)	49 (28.0)	0.3
Single/divorced/single	13 (7.4)	8 (4.6)	9 (5.1)	
Economic status, n (%)*				
High/average	18 (10.3)	22 (12.6)	21 (12.0)	0.5
Low	40 (22.9)	37 (21.0)	37 (21.0)	
Supplement use and medical history n (%)*				
Dietary supplement use	33 (18.9)	36 (20.6)	42 (24.0)	0.08
Family history of depression	14 (25.7)	15 (25.7)	15 (25.7)	0.8
CVD	25 (14.3)	16 (9.1)	15 (8.6)	0.8
Metabolic disorders	12 (14.3)	21 (9.1)	20 (8.6)	0.1
*Anthropometric and physical activity measures*				
Height (cm)^†^	154.0 (6.5)	155.0 (6.5)	155.7 (7.6)	0.1
Weight (kg)^†^	72.7 (11.8)	74.0 (12.6)	74.6 (12.3)	0.3
BMI (kg/m^2^)^†^	29.6 (8.4)	30.4 (5.5)	30.0 (4.7)	0.8
WC (cm)^†^	103.0 (13.6)	102.5 (14.0)	106.0 (13.6)	0.5
Physical activity (MET min/week)^†^	460.0 (462.0)	471.0 (789.0)	585.7 (1128.0)	0.02
*Dietary factors*				
Energy intake (kcal/day)^‡^	2369.4 ± 397.6	2138.4 ± 450.6	2324.6 ± 511.4	0.5
Carbohydrate (g/day)^†^	331.6 (110.7)	306.6 (103.4)	349.8 (108.4)	0.2
Protein (g/day)^‡^	83.4 ± 17.5	77.2 ± 17.0	84.9 ± 20.7	0.6
Fat (g/day)^†^	74.6 (32.6)	67.6 (17.6)	67.0 (33.1)	0.01
Carbohydrate (%EI)	57.4 ± 5.8	60.3 ± 5.4	61.5 ± 4.9	< 0.001
Protein (%EI)	14.0 ± 1.9	14.5 ± 1.8	14.6 ± 1.8	0.1
Fat (%EI)	29.5 (8.7)	27.3 (6.0)	27.5 (6.4)	0.003
*DASS-42 score*				
Depression^†^	8.5 (10.0)	6.0 (8.0)	5.5 (9.0)	0.03
Stress^†^	9.0 (9.0)	10.0 (9.0)	9.0 (10.0)	0.4
Anxiety^†^	8.0 (6.0)	7.0 (6.0)	5.0 (5.0)	0.02

Socioeconomic status defined as: having 3 or less living items for low status, 4–6 living items for average status, and 7–9 living items at home for high status

*DTAC* dietary total antioxidant capacity, *CVD* cardiovascular disease, *BMI* body mass index, *WC* waist circumference, *MET* metabolic equivalents task, *EI* energy intake, *DASS* depression, anxiety and stress scale

^‡^Data are expressed as mean ± SD

^†^Data are expressed as median (IQR)

* Number of subjects having the characteristic with %

** ANOVA test used for continuous variables and chi-square test used for categorical variables

According to partial correlation adjusted for energy intake, DTAC was highly correlated with the most of the dietary antioxidant components including vitamin C, β-cryptoxanthin, β-carotene, α-tocopherol, lycopene, lutein, zinc, magnesium (*P*-value < 0.04), but not with selenium. While serum TAC showed no significant correlation with these antioxidants, it indicated a moderate positive correlation with selenium (*r* = 0.14; *P*-value = 0.08) (Table 2).

**Table 2:** Correlation of DTAC and serum TAC value with intakes of some dietary antioxidant component

Dietary antioxidant intake	Partial correlations (*n* = 175)^†^			
	DTAC		Serum TAC	
	*r*	*P*	*r*	*P*
Vitamin C (mg/day)	0.53	< 0.001	0.03	0.6
β-Cryptoxanthin (μg/day)	0.43	< 0.001	0.02	0.7
β-Carotene (μg/day)	0.31	< 0.001	0.01	0.8
α-Tocopherol (mg/day)	0.18	0.01	0.07	0.3
Lycopene (μg/day)	0.15	0.04	0.04	0.5
Lutein (μg/day)	0.20	0.007	0.08	0.2
Zinc (mg/day)	0.16	0.03	0.01	0.8
Selenium (μg/day)	0.04	0.5	0.14	0.06
Magnesium (mg/day)	0.41	0.001	0.11	0.1

*DTAC* dietary total antioxidant capacity, *TAC* total antioxidant capacity

^†^ Partial correlations between DTAC and serum TAC. Dietary antioxidants intakes were adjusted for energy

As indicated in Table 3, after controlling for the potential confounders including age, time since menopause, education (year), WC (cm), physical activity (MET-min/week), dietary supplement use (yes/no), dietary intake of fiber (g/day), energy (Kcal/day) and coffee (g/day) in multivariable linear regressions, DTAC was inversely associated with depression (β = − 0.16, *P*-value = 0.03), anxiety (β = − 0.21, *P*-value = 0.007), but not stress (β = − 0.10, *P*-value = 0.1). We ran the same multiple regression for the women with family history of depression (n = 44), and found no significant association between DTAC and depression, stress and anxiety (data not shown).

**Table 3:** Multivariate linear regression analyses of the energy-adjusted DTAC and depression, stress, and anxiety scores (*n *= 175)

DTAC (μmol/day)^†^	Depression score		Stress score		Anxiety score	
	Coefficient standard β	P	Coefficient standard β	P	Coefficient standard β	P
Unadjusted model	− 0.14	0.053	− 0.05	0.3	− 0.19	0.008
Model 1	− 0.14	0.06	− 0.06	0.3	− 0.19	0.01
Model 2	− 0.16	0.03	− 0.10	0.1	− 0.21	0.007

Depression, anxiety, stress scores were log10 transformed

*DTAC* dietary total antioxidant capacity

Model 1, adjustment for age (year) and years since menopause (year)

Model 2, further adjustment for education (year), waist circumference (cm), physical activity (MET min/week), dietary supplement use (yes/no), dietary intake of fiber (g/day), energy (kcal/day) and coffee (g/day)

^†^DTAC was adjusted for energy intake by residual method

Multiple linear regression with the same covariates presented in Table 3 showed no association between serum TAC and depression (β = 0.04, *P*-value = 0.5), stress (β = 0.07, *P*-value = 0.3) and anxiety score (β = 0.08, *P*-value = 0.3). MDA level also had no relationship with depression (β = 0.05, *P*-value = 0.4), stress (β = − 0.008, *P*-value = 0.9) and anxiety score (β = 0.04, *P*-value = 0.5). There was no significant association between SOD and depression (β = − 0.03, *P*-value = 0.6), stress (β = − 0.06, *P*-value = 0.4) and anxiety (β = − 0.002, *P*-value = 0.9). Ox-LDL also failed to exhibit any association with depression (β = − 0.03, *P*-value = 0.6), stress (β = 0.06, *P*-value = 0.4) and anxiety score (β = 0.03, *P*-value = 0.6) (data not shown).

Adjusted mean values of serum oxidative stress biomarkers across the tertiles of DTAC are shown in Table 4. There was a significant difference between the adjusted mean of serum level of MDA (*P*-value < 0.001) and TAC (*P*-value = 0.04) across the tertiles of DTAC. The lowest adjusted mean MDA was observed at the third tertile of DTAC, while the women at second tertile of DTAC had the highest adjusted mean of serum TAC. No significant relationship was found for ox-LDL and SOD.

**Table 4:** Adjusted mean values for serum biomarkers of oxidative stress across tertiles of energy-adjusted DTAC (*n *= 175)

Variables	Tertiles of energy-adjusted DTAC							
	T1 (*n *= 58)		T2 (*n *= 59)		T3 (*n *= 58)		P-value*	Effect size of ANCOVA test
	Mean	95% CI	Mean	95% CI	Mean	95% CI		
TAC (mM)	0.44	0.42–0.45	0.46	0.44–0.47	0.44	0.42–0.45	0.04	0.03
MDA (µM)	5.3	4.7–6.0	4.8	4.2–5.4	3.4	3.0–3.8	< 0.001	0.13
Ox-LDL (ng/l)	1548.8	1339.6–1794.7	1655.7	1432.1–1918.6	1489.3	1285.2–1725.8	0.6	0.03
SOD (U/ml)	36.0	34.1–38.1	33.8	32.0–35.8	33.9	31.9–35.7	0.1	0.02

Values are geometric means and 95% CI

*DTAC* dietary total antioxidant capacity, *TAC* total antioxidant capacity, *MDA* malondialdehyde, *Ox-LDL* oxidized low-density lipoprotein, *SOD* superoxide dismutase

* ANCOVA test, adjusted for age, time since menopause, education (year), waist circumference (cm), physical activity (MET min/week), dietary supplement use (yes/no), dietary intake of fiber (g/day), energy (kcal/day), and coffee (g/day)

**Fig. 1:**
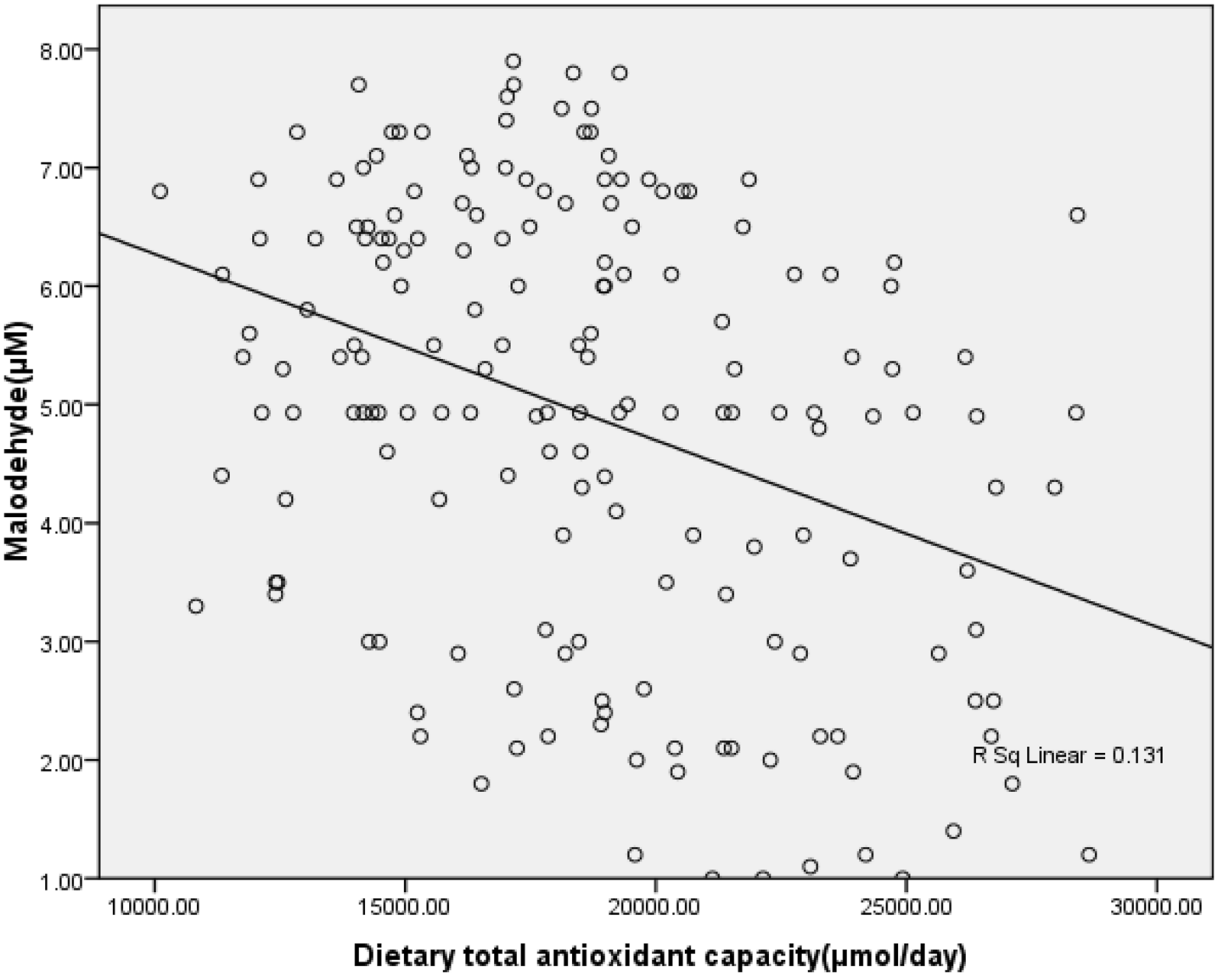
Correlation between dietary total antioxidant capacity (DTAC) and malondialdehyde (MDA) (r = − 0.36, P-value < 0.001)

Serum MDA showed an inverse correlation with DTAC (Fig. 1), whereas there was no correlation between DTAC and serum TAC, ox-LDL and SOD (data not shown). Multiple linear regression showed that the association between DTAC and serum TAC (β = 0.006, *P*-value = 0.9), ox-LDL (β = − 0.04, *P*-value = 0.5) and SOD (β = − 0.13, *P*-value = 0.07) was insignificant; whereas DTAC was inversely associated with serum MAD level (β = − 0.37, *P*-value < 0.001) as shown in Table 5.

**Table 5:** Multivariate linear regression analyses of the energy-adjusted DTAC and serum biomarkers of oxidative stress (*n *= 175)

DTAC (μmol/day)^†^	Coefficient standard β	P-value
TAC (mM)	0.006	0.9
MDA (µM)	− 0.37	< 0.001
Ox-LDL (ng/l)	− 0.04	0.5
SOD (U/ml)	− 0.13	0.07

Adjusted for age (year) and years since menopause (year) education (year), waist circumference (cm), physical activity (MET min/week), dietary supplement use (yes/no), dietary intake of fiber (g/day), energy (kcal/day) and coffee (g/day)

Serum biomarkers of oxidative stress were log10 transformed

*DTAC* dietary total antioxidant capacity, *TAC* total antioxidant capacity, *MDA* malondialdehyde, *Ox-LDL* oxidized low-density lipoprotein, *SOD* superoxide dismutase

^†^DTAC was adjusted for energy intake by residual method

## Discussion

To the best of our knowledge, the present study is among the few studies investigating the association between DTAC and mental disorders, and is the first study to examine this association among the postmenopausal women. After adjusting for multiple variables, we found an inverse association between DTAC and depression and anxiety scores. However, no association was detected between DTAC and stress score. In respect of oxidative status, serum MDA level was inversely associated with DTAC. Although, there was no linear relationship between serum TAC and DATC, subjects in the second tertile of DTAC had higher serum TAC level compared with the subjects in the first tertile. No association was observed between DTAC and serum ox-LDL and SOD concentration.

In line with our findings, a study on postmenopausal women revealed a strong negative link between the whole plant dietary pattern (consisting of whole grains, fruits, and vegetables) and depression [31]. Several studies also showed that adherence to healthy dietary patterns such as Mediterranean-type diet and Alternative Healthy Eating Index (AHEI) was associated with reduced risk of depression [32, 33] and anxiety [34]. However, a Brazilian study reported no association between DTAC and depression and anxiety [35]. These inconsistencies could be explained by various food processing and cooking in different cultures, which can affect the availability and content of dietary antioxidants [36].

In our study, no significant association was found between DTAC and depression, anxiety and stress in women with family history of depression. This may suggest that high TAC diets may not be as protective in individuals with genetic components of depression as it is in women without family history, although it needs to be confirmed in a larger study.

In the current study, no association was detected between DTAC and stress score. However, the result of other studies reported Mediterranean diet or appropriate consumption of fruits and vegetables (five servings/day) were associated with reduced risk of psychological distress [34, 37]. Further interventions are required to elucidate the effect of DTAC in relation to stress symptoms.

We found that women with higher DTAC had lower serum MDA level. Moreover, participants in the second tertile of DTAC had higher serum TAC level, while the level did not change in the third tertile, suggesting the existence of threshold levels associated with DTAC. However, serum TAC had no association with DTAC in multivariable model. These findings may suggest that high TAC diets may prevent lipid peroxidation, independent of serum TAC in postmenopausal women. So far, studies on the association between DTAC and blood antioxidant status among postmenopausal women and geriatric populations are scare and controversial [38–40]. A study in overweight and obese postmenopausal women reported that plasma TAC was positively associated with DTAC and dietary intake of antioxidant vitamins such as flavones and proanthocyanidins [38]. Proanthocyanidins and catechins are among the main compounds responsible for the increase of plasma TAC [41]. Notably, tea as the major food source for these compounds [42] was accounted for about 36% of total DTAC estimated in our study, followed by fruit (30%) and vegetable (14%) (data not shown); therefore, consuming more tea and fruits in the subjects with higher DTAC might have resulted in somehow elevated serum TAC and reduced MDA level. However, no association was found between DTAC with plasma TAC, MDA, ox-LDL level and antioxidant enzymes such as SOD in overweight and obese postmenopausal women [40], and elderly subjects [43]. Similarly, consumption of a high TAC diet for 2 weeks did not significantly alter the plasma TAC, ox-LDL, and protein carbonyls compared to low TAC diet in older people, although serum MDA reduced during low TAC diet. The short duration of intervention and compensatory response by homeostatic mechanisms was described for the lack of association [39]. Nevertheless, DTAC was negatively linked with plasma ox-LDL in young adults [30], suggesting that habitual high TAC diet might not be efficient in the substantial modification in ox-LDL in postmenopausal women. These discrepancies could be explained by many factors such as various food processing and cooking which can affect the availability and content of dietary antioxidants, as we mentioned before [36]. In addition, differences in bioavailability or absorption of antioxidants may affect blood TAC level [43]. Furthermore, it is possible that other components of blood antioxidants such as carotenoids may show better association with DTAC [44]. Some experimental studies found no significant increase in TAC level after intake of antioxidant-rich foods, while single carotenoids significantly increased [45, 46]. However, this information was not collected in the present study. Future studies are needed to clarify the effect of DTAC on various blood antioxidants in the menopause age.

The anti-oxidative and anti-inflammatory essence of the antioxidant-rich diets might explain the inverse association between DTAC and depression and anxiety. It is documented that depressive mood is highly related with impaired antioxidant defense and inflammatory status [47, 48]. Additionally, age-related reduction in the level of antioxidants has been shown in the frontal lobe of brain that is vital in mental functions [49, 50].

In our study, serum biomarkers were not associated with psychological factors. Plasma TAC and some other markers of oxidative stress were not associated with anxiety and depression [35]. However, a study in university male students found a negative correlation between serum TAC level and depression score [19]. Measurements of a few oxidative stress biomarkers may not truly reflect the in vivo antioxidant status and investigating various biomarkers has been suggested [51].

The present study has strengths and limitations. First, this study is the first study that explored association between DTAC and mental health in postmenopausal women. Second, to achieve the more accurate DTAC estimation, we assessed the intake of spices and herbs with high TAC value that are frequently used in Iranian diet. The under/overestimation in DTAC assessment due to cultivation procedures, storage, and cooking or some unmeasured confounding factors could have mediated the observed association [36]. Finally, cross-sectional design of study precluded us from concluding any cause–effect relationship.

Our findings suggest that higher DTAC is associated with lower depression and anxiety scores in postmenopausal women. In addition, higher DTAC is associated with lower serum MDA; hence postmenopausal women who are more prone to oxidative stress can reap the benefits of high TAC diets to improve their psychological disturbances and reduce the oxidative damage in their body.
